# Unveiling the Impact of Electronic Cigarettes (EC) on Health: An Evidence-Based Review of EC as an Alternative to Combustible Cigarettes

**DOI:** 10.7759/cureus.56451

**Published:** 2024-03-19

**Authors:** Sanjeev B Khanagar, Farraj AlBalawi, Aram Alshehri, Mohammed Awawdeh, Kiran Iyer, Laliytha Kumar Bijai, Ali Aldhebaib, Oinam Gokulchandra Singh

**Affiliations:** 1 King Abdullah International Medical Research Center, King Saud Bin Abdulaziz University for Health Sciences, Ministry of National Guard Health Affairs, Riyadh, SAU; 2 Preventive Dental Science Department, College of Dentistry, King Saud Bin Abdulaziz University for Health Sciences, Riyadh, SAU; 3 Restorative and Prosthetic Dental Sciences Department, College of Dentistry, King Saud Bin Abdulaziz University for Health Sciences, Riyadh, SAU; 4 Maxillofacial Surgery and Diagnostic Sciences Department, College of Dentistry, King Saud Bin Abdulaziz University for Health Sciences, Riyadh, SAU; 5 Radiological Sciences Program, College of Applied Medical Sciences, King Saud Bin Abdulaziz University for Health Sciences, Riyadh, SAU

**Keywords:** addiction, smoking, initiation, impact, health, electronic cigarettes, consequences, combustible cigarettes

## Abstract

Cigarette smoking has been considered a major public health concern due to its serious impact on health. However, smokers intending to quit may find long-term abstinence challenging. When smoking an electronic cigarette (EC), users can experience a sensation and taste similar to that of smoking a combustible cigarette. Therefore, manufacturers promote these products as a viable substitute for combustible cigarettes. However, several researchers report the serious health impacts experienced by EC users. Therefore, this review aims to examine the health impacts of EC use. Based on the findings of the research papers reported in the literature, the role of EC as a smoking cessation tool is unclear. Several researchers have also reported a significant association between EC usage among non-smokers at baseline and the future initiation of combustible cigarette smoking. EC use significantly impacts user health. The nicotine that is present in EC e-liquids can elevate blood pressure, resulting in blood vessel constriction and an increase in heart rate, ultimately leading the body to an ischemic condition, resulting in myocardial infarction (MI), stroke, and increased arterial stiffness. Researchers report a higher likelihood of prediabetes among EC users; its usage was associated with higher OR of having asthma attacks and higher OR of reporting depression and has an impact on birth outcomes among pregnant women. Men using EC are more likely to report erectile dysfunction than non-users. EC also has a significant impact on oral health, which includes periodontal diseases, mucosal lesions, irritation in the mouth and throat, reduced salivary flow, and an increased risk of developing cancer. The physical injury resulting from exploding EC is another health concern. The frequently burned areas included the hands, face, genitalia, and thighs. Marketers promote EC as an alternative to combustible cigarettes and a tool for quitting smoking. However, the Food and Drug Administration has not approved them for smoking cessation. EC can have a serious impact on the health of their users; hence, the findings of this paper have several implications, including the need for regulation of the sales and marketing of these products and educating the users on the impact of these products on their health and safety.

## Introduction and background

For many decades, smoking has been considered one of the major public health concerns due to its serious impact on health. Researchers have estimated that around eight million smokers die prematurely due to smoking [1]. Reports state that over 1 million non-smokers exposed to second-hand smoke die every year [1]. Cigarette smoke is comprised of many harmful chemicals that are known to cause cancer, which makes them directly responsible for death related to lung cancer and chronic lung diseases [2]. Smoking affects every organ of the human body, which is known as an etiological factor for chronic obstructive pulmonary disease and a cause of lung cancer. It is also a known cause of coronary heart disease and stroke [3]. Smoking also has negative social consequences, such as limited social interactions. Non-smokers, especially children, when exposed to cigarette smoke can experience health impacts, which include reduced lung function, the risk of developing asthma, and the risk of developing middle-ear infections [1].

Considering these health consequences, quitting smoking is the best way to reverse the health benefits. However, smokers intending to quit may find long-term abstinence challenging. Researchers have reported on the difficulties and the increased relapse chances among smokers [4]. Several researchers report that behavioral support along with replacing nicotine as a replacement therapy in the form of nicotine gums or patches can help in addressing withdrawal symptoms; however, long-term quit rates remain the same [5-7]. Despite substituting nicotine, there still exists a major limitation, which is the lack of sensory, social, and behavioral aspects of smoking (holding cigarettes and taking puffs) [8]. This limitation can be addressed using electronic cigarettes (EC), which could not only act by relieving the unpleasant nicotine withdrawal symptoms but can also act as an alternative to combustible cigarettes by addressing the smoking behavior and the sensations felt with smoking [9,10]. EC can provide a sensation and taste similar to those experienced when smoking a combustible cigarette [11]. Considering the advantages of EC, these products are marketed as an acceptable alternative to combustible cigarettes [12,13]. Hence, the aim of this review article is to report on the health impacts of EC use.

## Review

Literature search criteria

Several databases such as PubMed, Scopus, Embase, Cochrane, Web of Science, Google Scholar, and Saudi Digital Library were searched for articles associated with this review. The following keywords were used along with the appropriate Boolean operators, EC, e-cigarette, vaping, vape, smoking, combustible cigarette, impact, effect, health, blood vessels, dementia, depression, erectile dysfunction, pregnancy, respiratory, pulmonary function, lungs, cardiovascular health, stroke, neurovascular effects, hypertension, blood pressure (BP), diabetes and oral health. A data search was performed for the articles that were published until 30 January 2024. On the initial search, 158 articles were reflected, and then the articles were filtered after completely reviewing their content, following which 78 articles were considered for final reporting. We looked for original research articles, review articles, systematic reviews, and meta-analysie as there was no limit set for the study design for the included articles.

Do EC help in quitting combustible cigarette smoking?

Marketers usually promote EC as an alternative product or as an aid for smokers trying to restrain from combustible cigarettes. Kalkhoran et al. included 38 studies in their systematic review, conducting sensitivity and random effects analyses on all 20 studies with control groups. EC users had 28% lower odds of quitting smoking when compared to never-users. Studying smokers who use EC regardless of their motivation to stop did not significantly alter the association between EC use and quitting [14]. Wang et al. conducted a meta-analysis to determine the association between EC use and smoking cessation. Out of the 64 publications found, 55 were observational studies, and nine were randomized clinical trials (RCTs). These studies did not find a link between EC use and quitting [15].

Malas et al., who conducted a systematic review that aimed to assess the efficacy of EC as a cessation device, reported that most of the included research discovered that, in lab settings, EC, particularly second-generation models, could reduce cravings and symptoms associated with smoking. However, the Grading of Recommendations, Assessment, Development, and Evaluations (GRADE) approach determined that the evidence supporting the effectiveness of EC in helping smokers quit was very low to low, while the evidence supporting the reduction of smoking was very low to moderate [16].

Hence, considering the findings of these evidence-based research papers, the role of EC as a smoking cessation tool is unclear.

Do EC play a role in the initiation of combustible cigarette smoking?

The use of EC among youngsters who have never smoked is rising in many countries [17,18], and the main reasons for this usage among young people are experimentation and curiosity, rather than quitting smoking. There are concerns that individuals who have never smoked before may try combustible tobacco cigarettes and eventually start smoking regularly if they use EC, particularly young adults and children [19-21]. Baenziger et al. reported on a meta-analysis that aimed to assess the relationship between EC usage and the subsequent initiation of combustible tobacco cigarette smoking among non-smokers [22]. The researchers reported that all 25 research articles included in their meta-analysis revealed that exposure to EC increased the likelihood of smoking initiation. When comparing EC users to non-users using a random-effects model, the OR ratio for smoking initiation among never-smokers at baseline was 3.19, whereas the OR for current smoking among non-smokers at baseline was 3.14. Smoking relapse was more common among ex-smokers who used EC than those who did not (OR: 2.40). The study concluded that nonsmokers who use EC have a higher likelihood of starting to use combustible cigarettes and becoming regular smokers than those who do not use them [22].

Chan et al. conducted another meta-analysis to evaluate the relationship between EC use at baseline and the initiation of combustible cigarette smoking at follow-up among adults aged less than 18 years [23]. The findings of this meta-analysis, which included 11 research articles, concluded that there is a significant longitudinal association between vaping and smoking (OR: 2.93) [23].

O'Brien et al. conducted another meta-analysis to evaluate the link between EC use among teenagers at baseline with subsequent initiation of combustible cigarette smoking at follow-up [24]. The findings of this meta-analysis were that the teenagers who had ever used EC at baseline had an OR of 4.6 of initiating combustible cigarette smoking, concluding that EC usage is an important health-related harm [24].

Therefore, the findings of the above-mentioned papers suggest a significant association between EC usage among non-smokers at baseline and the future initiation of combustible cigarette smoking.

What is the impact of EC usage on general health?

Studies have widely accepted EC as an alternative to combustible cigarettes and have reported them as safe by comparing their content and safety. However, EC users experienced equal or higher levels of formaldehyde and metal compounds compared to combustible cigarette users [25-27]. The heat induced by an EC causes oxidation and decomposition of glycols, leading to the generation of harmful constituents that are inhaled [28]. The vapors generated from EC also contain several metals, including cadmium, lead, nickel, chromium, tin, and silver [25]. Volatile organic compounds, such as benzene, styrene, ethylbenzene, and toluene, are known to trigger irritation and headaches and damage the liver, kidney, and central nervous system [25]. Butyraldehyde and aldehydes, such as formaldehyde, acetaldehyde, acetone, acrolein, and propionic aldehyde, are among the carbonyls found in EC. It is well-known that these compounds are extremely irritating and harmful [25].

EC usage cardiovascular health

Nicotine in EC e-liquids can elevate BP a few minutes after acute nicotine exposure, resulting in blood vessel constriction and an increase in heart rate and ultimately leading the body to an ischemic condition [29]. Blood vessel damage and ischemic organ damage (myocardial infarction (MI), stroke, and increased arterial stiffness) are all caused by a decreased ability to dilate vessels. All of these things raise the risk of mortality [30]. Siddiqi et al. conducted a meta-analysis to determine the relationship between EC exposure with cardiovascular health. Twenty-seven studies were evaluated in this meta-analysis, and it was found that, following acute EC exposure, heart rate dramatically increased. Significant elevations in both the diastolic and systolic BP were also reported. The authors concluded that there is a noteworthy correlation between smoking EC and elevated cardiovascular hemodynamic measurements and biomarkers [29]. Martinez-Morata et al., in their systematic review, reported the relationship of EC usage with BP endpoints [31]. The study included 13 EC cross-over design trials, along with one observation study. The authors concluded that EC usage may result in short-term elevations in both diastolic and systolic BP. Skotsimara et al. conducted a meta-analysis on the impact of EC and risks associated with the cardiovascular system, concluding that the evidence on the cardiovascular effects of EC is concerning [32].

EC usage and prediabetes

The prevalence of prediabetes is expected to affect over 470 million individuals globally by 2030 [33]. A study reported that having prediabetes increases the chance of developing cardiovascular diseases, such as peripheral vascular diseases, atherosclerotic disorders, and stroke [34]. Zhang et al. conducted a study to determine the relationship between EC and prediabetes. They reported that the OR for prediabetes were 1.22 for current EC users and 1.12 for former EC users when compared with never EC users [35]. The authors concluded that there was a higher likelihood of prediabetes in this sample of adult Americans who used EC [35]. Atuegwu et al. conducted another study to determine the relationship between EC usage and self-reported diagnosis of prediabetes among non-cigarette smokers [36]. The authors found that current users were more likely to report on having prediabetes (OR: 1.97) when compared to those who had never used EC [36].

EC usage and asthma

Asthma is a non-infectious chronic disease that is more prevalent among both children and adults. Currently, asthma affects approximately 300 million people worldwide, representing a significant global public health concern [37]. Research has linked conventional cigarettes to respiratory conditions, such as chronic obstructive pulmonary disease (COPD), chronic bronchitis, and asthma. Given the increasing popularity of EC, a meta-analysis reported on the association between EC exposure and the risk of asthma [38]. The authors reported that EC use in the past had a connection with asthma (OR: 1.22), and there was a substantial association between current EC use and asthma (OR: 1.30) [38]. Osei et al. studied the link between EC use and asthma in people who had never smoked a regular cigarette. They discovered that current EC users had 39% higher OR of self-reported asthma compared to people who had never used EC [39]. Lee et al. also reported an association between using EC and asthma among the US population. The study concluded that current usage of EC was associated with higher OR of having an asthma attack [40].

EC usage and ocular health

There is evidence linking tobacco use to a number of eye conditions, including diabetic retinopathy, cataracts, thyroid orbitopathy, and age-related macular degeneration [41-43]. Considering the higher usage of EC among the younger population, it is important to know the impact of EC smoking on ocular health. Kalayci et al. reported a study that aimed to assess the impact of EC smoking on retinal microvascular architecture. The authors reported that EC smoking causes enlargement of the foveal avascular zone area and decreases vascular densities [44]. Md Isa et al. conducted a study to investigate the effect of EC smoking on ocular surface health. The authors concluded that EC users displayed moderate-to-severe symptomatic dry eye and poorer tear film quality compared with nonsmokers [45]. Golla A. et al. conducted a study to assess the association between EC usage and visual impairment among the adult population in the United States. EC usage among the current users was significantly associated with higher OR of visual impairment in comparison with non-EC users [46].

EC usage and neurovasculature

Nicotine, the main ingredient in both EC and cigarettes, is also associated with vascular dysfunction. Additionally, early vascular dysfunction and blood-brain barrier (BBB) breakdown may be the underlying causes of various dementias, including Alzheimer's disease. Smoking is also a risk factor for the development of cerebral small vessel disease and Alzheimer's disease [47,48]. Nicotine is harmful to both macro- and micro-vascular systems. Inhaled vaporized nicotine acutely increases heart rate, BP, and arterial stiffness while hindering microvascular dilatation caused by prostaglandins [49]. Heldt et al. conducted a study to assess the impact of long-term EC exposure on BBB function in mice. The study concluded that, regardless of the amount of nicotine in the product, the prolonged use of EC may have a harmful impact on neurovascular health and might cause cognitive dysfunction. Using EC has effects that are similar to those of exposure to combustible tobacco, and in some situations, they may even be more harmful [50]. Awad et al. conducted a meta-analysis that aimed to assess the relationship between EC usage and the risk of stroke in comparison to non-smokers. The authors reported that usage was significantly associated with an increased risk of stroke (OR: 1.52) when compared with non-users [51].

EC usage and depression

With the popularity of EC, there is a high prevalence of their usage among the younger population. A study reported that the prevalence of EC use among those who reported having depression was 9.1% when compared with a general population prevalence of 4.5%. The authors also reported that former EC users had 1.60 times higher OR of reporting a history of clinical diagnosis of depression than never-users, in comparison with current EC users, who had 2.10 times higher OR. Furthermore, current EC users who reported daily use had 2.39 times higher OR of reporting depression, while those who reported occasional use had 1.96 times higher OR [52].

EC usage and its impact on the reproductive system

Several researchers report that smoking during pregnancy increases the health hazards that might result in the risk of preterm birth, low birth weight, and birth abnormalities [53,54]. Nicotine is one known element that causes abnormalities in a fetus's development [55]. With the rise of EC usage among the younger population, there is a huge concern for the health impact of these products, as the flavorings used in EC may be dangerous to a growing fetus [56]. Lin et al. conducted a study to determine the relationship between EC usage and its impact on birth outcomes among pregnant women. Mint or menthol-flavored EC usage was found to be correlated with a higher risk of fetus death (OR: 3.27), according to the study's conclusion [57]. Galbo et al. conducted a study to assess the association between the use of EC during pregnancy and unfavorable birth outcomes. The authors reported that the OR of unfavorable birth outcomes increased by 62% among women who reported EC use during pregnancy versus women who did not (adjusted odds ratio (AOR): 1.62)) [58]. Regan et al. reported on the impact of combustible and EC usage during pregnancy on pregnancy outcomes. The authors reported on a higher prevalence of low birth weight among those who used EC, when compared to women who quit smoking [59].

Several researchers report on the relationship between combustible cigarette smoking and the prevalence of erectile dysfunction, which is mainlydue to endothelial dysfunction [60-63]. El-Shahawy et al. conducted a study to determine the relationship between EC usage and erectile dysfunction. The authors concluded that the current daily users of EC were more likely to report erectile dysfunction than never users in both the full (AOR=2.24) and restricted (AOR=2.41) samples [64].

EC usage and oral health

EC usage also impacts oral health in addition to general health. According to recent systematic reviews, the most frequent side effects of EC on oral health are periodontal disease and irritation of the mouth and throat [65,66]. Deeper probing depths and greater accumulation of plaque are the most prevalent periodontal issues. EC aerosols can induce an oxidative stress response in oral keratinocytes that can be cytotoxic. Specific metals, such as nickel, lead, and chromium, have a greater concentration in EC aerosols than in traditional cigarettes, significantly impacting the gingival epithelium, periodontal ligament, and oral mucosa [67].

Irusa et al. reported on the relationship between using EC and the risk of developing dental caries. In comparison to the control group, the EC group had a statistically significant difference (P < 0.001) in caries risk levels [68].

Amanian et al. [69] conducted a scoping review where they analyzed 32 research papers to assess the impact of EC on the oral mucosa. The majority of the articles reported throat and mouth irritation, followed by cough, as the most common side effects of EC [70].

Xu et al. reported on the longitudinal clinical study to assess the effect of EC use on the bacterial structure in the saliva of patients with periodontitis. The study reported that, over time, EC smoking may change the bacterial makeup of saliva in a manner similar to that of cigarette smoking, which could increase the relative abundance of pathogens linked to periodontal disease. EC use has also been linked with increased levels of proinflammatory cytokines, such as tumor necrosis factor alpha (TNF-α) and interferon gamma (IFN-γ), exacerbating disease states [71]. Mohajeri et al. also reported on the relationship between EC usage and periodontal health among the US adult population. Regular EC users had increased probabilities of poor periodontal health, including loss of bone around teeth, compared to never EC users. Furthermore, EC use was independently linked with an elevated risk of oral health problems [72].

Guo et al. reported on a meta-analysis to assess the occurrence of xerostomia in a healthy group using combustible tobacco and/or EC. According to the authors, the prevalence of xerostomia among combustible cigarette users was 24%, whereas the prevalence among users of EC was 33% [73].

DLima et al. evaluated the effects of e-liquid on human oral squamous cell carcinoma (OSCC) cell lines (CAL27 and HSC3), mouse oral cancer cell lines (AT84), and normal oral epithelium (NOE) cell lines using assays for cell proliferation, survival/cell death, and cell invasion. The authors observed that e-liquid causes morphological alterations linked to increased motility and invasive characteristics, as well as encouraging the proliferation and anchorage-independent development of OSCC. Moreover, independent of the flavor of the EC, cells exposed to e-liquid exhibit noticeably decreased cell viability. The authors concluded that the capacity of e-liquid to cause proliferative and invasive characteristics, together with the activation of the epithelial-to-mesenchymal transition process, may facilitate the aggressive phenotype of pre-existing oral malignant cells and contribute to the development of cancer in normal epithelial cells [74].

EC usage and physical injuries

The physical injury resulting from exploding EC is another health concern. Batteries that burst in EC have the potential to burn users. Previous events imply that explosions could result from an overheating battery [75]. Seitz et al. reported a systematic review of burn injuries caused by EC explosions. Out of the 164 registered cases, the authors observed that EC explosions were most commonly seen in the pockets (65%), followed by the faces and hands of their users. Frequently burned areas included the hands, face, genitalia, and thighs. The most common burn severity was second-degree burns (35%) or a mix of second- and third-degree burns (20%) [76]. Kaltenborn et al. reported on a retrospective study to determine the patterns of EC-related injuries and their clinical management among 46 reported cases. The injury patterns included the groin region (69%), followed by hands (25%), and face (7%), and all of these required surgical management [77]. Boissiere et al. reported on cases of burns related to EC explosions among 16 patients, the characteristics of the burns, and their management. Each of them had burns of the second or third degree. Burns occurred on the hands, buttocks, thorax, thighs, and genital areas, and they were often associated with the placement of an EC in their pocket. Most of these cases required skin grafts and surgical management [78].

Recommendations

Considering the health impacts resulting from EC usage, these products should not be promoted as an alternative combustible cigarette. Health professionals should educate and motivate both combustible cigarette and EC users to quit smoking, by providing the users with all the information on health impacts related to smoking. The legislative bodies should emphasize stringent policies and guidelines to implement health warnings, information on the addictive nature of the products, and information reading the health impacts related to its usage and restrict the sale of the products to those who have attained the legal age. Considering the severe health consequences related to these products, several countries have banned the sale of these products. However, countries where it is considered a legal product should make strict policies on the sales of these products.

## Conclusions

EC manufacturers market these products as an alternative to combustible cigarettes and a device to be considered when making an attempt to quit smoking; however, the Food and Drug Administration has not approved them as a smoking cessation tool. Many users consider them safer than combustible cigarettes; thus, this paper aims to report on EC's health impacts. The current evidence suggests that EC use can lead to the subsequent initiation of combustible cigarette smoking among non-smokers. EC have a serious impact on the health of their users, which includes an impact on cardiovascular health, elevated levels of BP, stroke, an increased risk of developing prediabetes, increased chances of developing asthma, visual impairment, depression, fetal abnormalities among pregnant women, an increased risk of erectile dysfunction among men, and a significant impact on oral health. Oral health impacts include periodontal diseases, mucosal lesions, irritation in the mouth and throat, reduced salivary flow, and an increased risk of developing cancer.

Along with this, EC have numerous reports of explosions resulting mostly from the batteries, which can result in severe damage to things such as hands, faces, and genitals. This review has several implications, including the need for regulation of the sales and marketing of these products and educating the users on the impact of these products on their health and safety.
